# Pretracheal Lymph Node Subdivision in Predicting Contralateral Central Lymph Node Metastasis for Unilateral Papillary Thyroid Carcinoma: Preliminary Results

**DOI:** 10.3389/fendo.2022.921845

**Published:** 2022-07-18

**Authors:** Qiang Chen, Yang Liu, Wei Lu, Lingyun Zhang, Anping Su, Feng Liu, Jingqiang Zhu

**Affiliations:** Department of Thyroid and Parathyroid Surgery Center, West China Hospital, Sichuan University, Chengdu, China

**Keywords:** pretracheal lymph node, central neck dissection, papillary thyroid carcinoma, central lymph node metastasis, clinically node-negative neck

## Abstract

**Background:**

The aims of this study were to assess the clinical value of pretracheal lymph node subdivision in identifying patients with contralateral central lymph node metastasis (CLNM) and risk factors for occult contralateral CLNM in unilateral PTC.

**Methods:**

A total of 139 unilateral PTC patients with a clinically node-negative neck (cN0) who underwent bilateral central neck dissection (CND) were prospectively enrolled. Intraoperatively, the pretracheal region was further divided into ipsilateral and contralateral subregions. Ipsilateral and contralateral pretracheal lymph nodes (LNs) as well as other CLNs (prelaryngeal, ipsilateral paratracheal and contralateral paratracheal) were labeled separately and sent for pathological examination. Demographic and clinicopathologic variables were analyzed to identify factors predictive of contralateral CLNM.

**Results:**

Of 139 patients, bilateral CLNM was present in 37 (26.6%) patients. Contralateral pretracheal LNM was significantly associated with contralateral CLNM. In multivariate analysis, prelaryngeal LNM (*P* = 0.004, odds ratio = 3.457) and contralateral pretracheal LNM (*P* = 0.006, odds ratio = 3.362) were identified as risk factors for contralateral CLNM. Neither neck recurrence nor distant metastasis was observed within the mean follow-up duration of 9.1 ± 1.8 months.

**Conclusions:**

In most unilateral cN0 PTCs, performing ipsilateral CND is appropriate, while patients presenting with evident nodal disease intraoperatively or preoperatively in the contralateral central neck should undergo bilateral CND. Intraoperative re-evaluation of prelaryngeal and contralateral pretracheal LNs may be helpful in determining the extent of CND.

## Introduction

Despite the excellent overall prognosis, papillary thyroid carcinoma (PTC) is characterized by early regional lymph node metastasis (LNM), and the central neck compartment (level VI) is usually the first involved region. Central lymph node metastasis (CLNM) has been reported in 30%–90% of patients who undergo elective central neck dissection (CND) (1, 2). Ipsilateral CND is considered necessary for patients with suspicious nodal metastasis, but when to perform contralateral CND in unilateral PTC is controversial. Bilateral CND has been recommended by some endocrine surgeons because it can enable complete nodal dissection, which may reduce the risk of locoregional recurrence and distant metastasis (3–5). In fact, however, most patients with unilateral PTC only have ipsilateral CLNM without contralateral CLNM. On the other hand, bilateral CND is associated with a higher rate of surgical complications such as hypoparathyroidism and recurrent laryngeal nerve injury, even if it is performed in experienced hands (6, 7). Nonetheless, the risk of nodal metastasis and locoregional recurrence in the contralateral central neck cannot be neglected (8). It is important to understand the characteristics and prognosis of CLNM in unilateral PTC. Therefore, accurate assessment of patients at high risk of CLNM, especially contralateral CLNM, may facilitate determining the appropriate extent of CND, which could decrease unnecessary morbidities caused by bilateral CND.

Pretracheal lymph nodes (LNs) have been found to be closely connected with ipsilateral/contralateral central lymph nodes (CLNs) (9). Although some studies have investigated the risk factors for contralateral CLNM, there have been few studies evaluating whether pretracheal LNs could be used as sentinel LNs that aid in identifying patients with occult contralateral CLNM. In this study, we hypothesized that pretracheal LN subdivision could be of value for the identification of PTC patients with occult contralateral CLNM.

The aims of the present study were to evaluate the clinical value of pretracheal LN subdivision to aid in surgical decision-making regarding the extent of CND and risk factors for occult contralateral CLNM in unilateral PTC.

## Materials and Methods

### Patient Population

From October 2019 to December 2020, 193 patients consented to undergo pretracheal LN subdivision, total thyroidectomy and bilateral CND for the treatment of PTC in our institution. All patients had a preoperative diagnosis of PTC, and no suspicious neck LNs were detected by palpation or ultrasound (US) perioperatively (cN0). Patients with the following were excluded from this study: history of previous thyroidectomy, non-PTC carcinoma, PTC variants, isthmus tumor, bilateral tumors, and lateral neck dissection. Finally, 139 patients were prospectively enrolled in this study. This study was approved by the Institutional Review Board of West China Hospital, Sichuan University (IRB-HX2019012), and all patients provided written informed consent.

### Surgical Procedures

**Figure 1:**
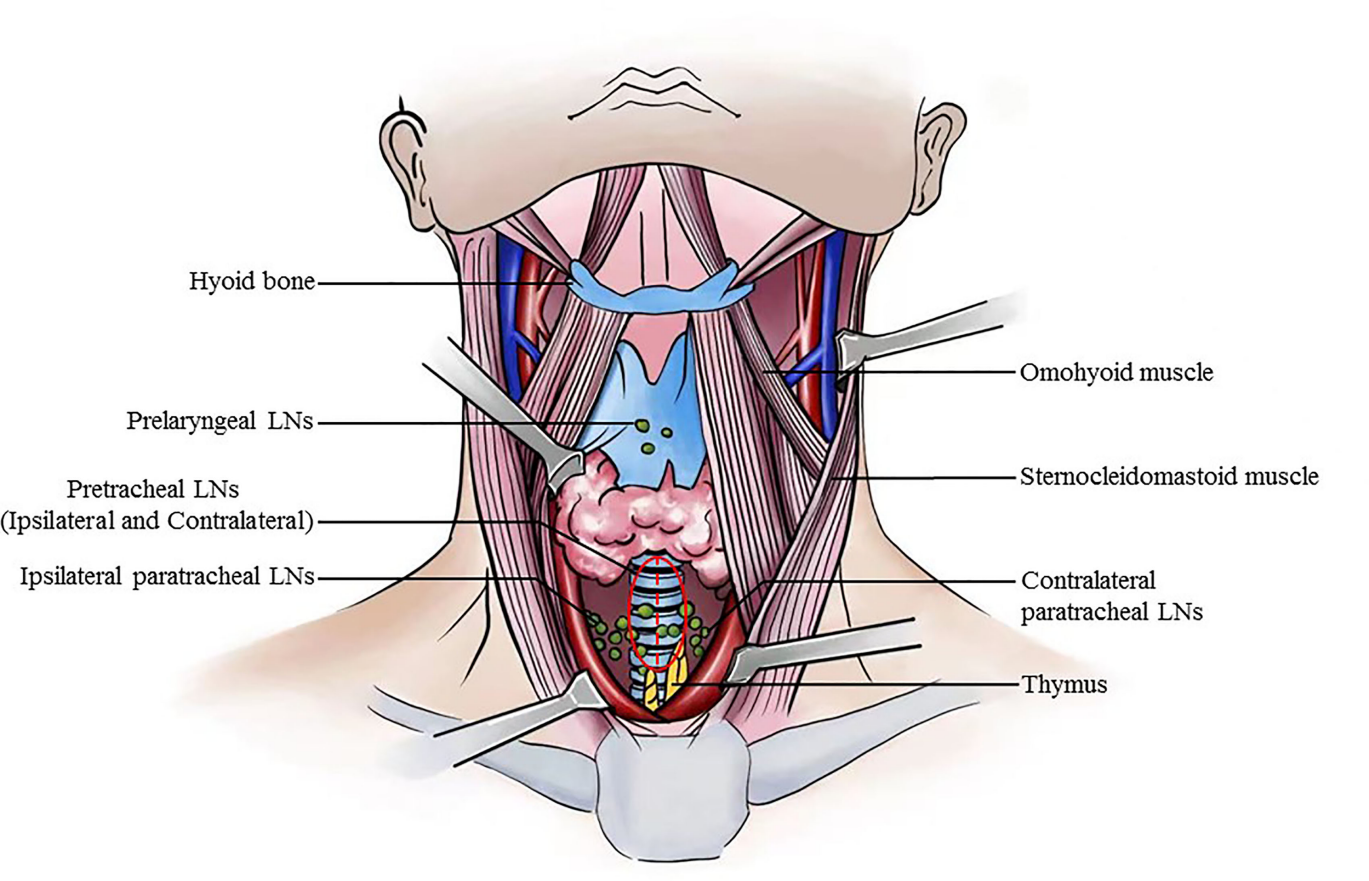
Illustration of pretracheal lymph node subdivision in the central compartment.

A standardized surgical procedure was carried out in the present study to minimize morbidities and ensure comprehensive removal of LNs in the central compartment. CND was defined as a level VI dissection extending superiorly to the hyoid bone, inferiorly to the suprasternal notch, laterally to the carotid sheaths, and dorsally to the prevertebral fascia. Bilateral CND included the removal of prelaryngeal, pretracheal, and both the paratracheal nodes on the side of the tumor and contralateral to the tumor (10). Moreover, pretracheal LNs are further divided into ipsilateral pretracheal LNs and contralateral pretracheal LNs intraoperatively based on the middle line of the anterior wall of the trachea (**Figure 1**).

### Histopathological Examination of Surgical Specimens

LNs in each subsite of the central compartment were separated and labeled after dissection and sent for pathological examination along with the resection thyroid specimens. Thyroid specimens and dissected LNs were microscopically examined by two or more experienced pathologists. The following factors were assessed: primary tumor size, multifocality, capsular invasion, extrathyroidal extension (ETE), chronic lymphocytic thyroiditis, the number of metastatic LNs, and the total number of LNs in each subsite. The pathological stage of thyroid cancer was determined in accordance with the 8^th^ edition of the American Joint Committee on Cancer Staging Manual (11).

### Postoperative Management and Follow-Up

Postoperative serum calcium and intact parathyroid hormone concentrations were measured in all patients. Patients who developed hypocalcemia were treated with oral calcium and vitamin D supplements, and those who developed significant symptoms were administered intravenous calcium gluconate. Transient hypocalcemia was defined as an ionized serum calcium concentration of <2.1 mmol/L and the calcium level recovered to normal within 6 months, while permanent hypocalcemia was defined as an ionized serum calcium concentration that remained below normal at ≥6 months after surgery and required oral calcium and vitamin D supplementation. Preoperative fibrolaryngoscopic examination was performed for each patient, while postoperative examination was selectively performed in the case who developed hoarseness after surgery. Vocal cord palsy that lasted for 6 months postoperatively was regarded as permanent. All patients received thyroid-stimulating hormone suppression therapy after surgery and underwent regular follow-up at 6-to 12-month intervals with clinical evaluation including serum thyroglobulin (Tg) level, ultrasonography (US) and CT. Locoregional recurrence was defined as the presence of tumors or metastatic LNs on cytological diagnosis. Radioactive iodine (RAI) therapy was performed based on tumor stage and risk factors, in accordance with the American Thyroid Association guidelines (10).

### Statistical Analysis

Statistical analyses were performed using SPSS software (version 19.0, IBM Corp, Armonk, NY, USA), and a *P* value <0.05 was considered to be statistically significant. Continuous variables are presented as the mean ± standard deviation (SD), and categorical variables are presented as numbers with percentages (%). Student’s t-test was used for continuous variables, and the chi-square test was used for categorical variables in univariate analysis. Multivariate analysis was performed by binary logistic regression to determine if the clinicopathological characteristics were risk factors for contralateral CLNM.

## Results

### Clinicopathological Characteristics of 139 PTC Patients Who Underwent Total Thyroidectomy With Bilateral CND

The demographics and clinical data of the 139 patients (34 men and 105 women) are summarized in **Table 1**. The median age was 39 years (range, 18–72 years). Of these 139 patients, 89.9% (125/139) were <55 years old, and 10.1% (14/139) were ≥55 years old. The mean ± SD size of the primary tumor was 1.3 ± 0.7 cm (range, 0.6–3.7 cm). Fifty-six (40.3%) patients had a primary tumor ≤1 cm, and 83 (59.7%) patients had a primary tumor >1 cm. Multifocality was observed in 33 (23.7%) patients, capsular invasion was found in 66 (47.5%) patients, and ETE was confirmed in 31 (22.3%) patients. The mean ± SD number of dissected nodes was 5.4 ± 3.2 in the ipsilateral paratracheal region, 3.8 ± 3.0 in the contralateral paratracheal region, 1.4 ± 1.2 in the prelaryngeal region, and 3.8 ± 2.2 in the pretracheal region. One hundred and three (73.4%) patients had pretracheal LNM, 47 (33.8%) patients had prelaryngeal LNM, 88 (63.3%) patients had ipsilateral CLNM, and 37 (26.6%) patients had bilateral CLNM. Among these patients, 7 (5.0%) patients had isolated contralateral CLNM.

**Table 1:** Clinicopathologic characteristics of patients.

Variables	
Total number of patients	139
Age, median, yrs	39 (18–72)
<55 y/≥55 y, *n* (%)	125 (89.9)/14 (10.1)
Gender, *n* (%)	
Female	105 (75.5)
Male	34 (24.5)
Primary tumor size, cm	1.3±0.7 (0.6–3.7)
≤1 cm/>1 cm, *n* (%)	56 (40.3)/83 (59.7)
Multifocality, *n* (%)	33 (23.7)
Chronic lymphocytic thyroiditis, *n* (%)	43 (30.9)
Capsular invasion, *n* (%)	66 (47.5)
Extrathyroidal extension, *n* (%)	31 (22.3)
Prelaryngeal LNM, *n* (%)	47 (33.8)
Pretracheal LNM, *n* (%)	102 (73.4)
Ipsilateral pretracheal LNM, *n* (%)	73 (52.5)
Contralateral pretracheal LNM, *n* (%)	62 (44.6)
Ipsilateral CLNM, *n* (%)	88 (63.3)
Contralateral CLNM, *n* (%)	37 (26.6)
CLNM, central lymph node metastasis; LNM, lymph node metastasis.	

CLNM, central lymph node metastasis; LNM, lymph node metastasis.

### Risk Factors for Contralateral CLN Metastasis in 139 PTC Patients

In univariate analysis, prelaryngeal LNM (*P =* 0.002) and ipsilateral paratracheal LNM (*P =* 0.009) were significantly associated with contralateral CLNM (**Table 2**). Moreover, contralateral pretracheal LNM (*P =* 0.001) was significantly associated with contralateral CLNM, while pretracheal LNM was not associated with contralateral CLNM. Among patients with central lymph node metastasis, contralateral pretracheal lymph node positivity was significantly associated with patient age (*P =* 0.034) (**Table 3**). In multivariate analysis, prelaryngeal LNM (*P* = 0.004, odds ratio = 3.457) and contralateral pretracheal LNM (*P* = 0.006, odds ratio = 3.362) were independent predictive factors for contralateral CLNM (**Table 4**).

**Table 2:** Univariate analysis of clinicopathologic characteristics for contralateral CLNM.

	Contralateral CLNM, *n* (%)	*P*
Age, yrs		1.000*
<55	33 (26.4)	
≥55	4 (28.6)	
Gender		0.118
Female	25 (23.8)	
Male	12 (35.3)	
Primary tumor size, cm		0.126
≤1	11 (19.6)	
>1	26 (31.3)	
Capsular invasion		0.291
Yes	22 (33.3)	
No	10 (23.8)	
Extrathyroidal extension		0.422
Yes	5 (16.1)	
No	10 (23.8)	
Multifocality		0.922
Yes	9 (27.3)	
No	28 (26.4)	
Chronic lymphocytic thyroiditis		0.289
Yes	14 (32.6)	
No	23 (24.0)	
Prelaryngeal LNM		0.002
Yes	20 (42.6)	
No	17 (18.5)	
Pretracheal LNM		0.216
Yes	30 (29.4)	
No	7 (18.9)	
Ipsilateral CLNM		0.009
Yes	30 (34.1)	
No	7 (13.7)	
Ipsilateral pretracheal LNM		0.17
Yes	23 (31.5)	
No	14 (21.2)	
Contralateral pretracheal LNM		0.001
Yes	25 (40.3)	
No	12 (15.6)	

CLNM, central lymph node metastasis; LNM, lymph node metastasis. *Fisher’s exact test.

**Table 3:** Characteristics of contralateral pretracheal lymph node positivity in patients with contralateral CLNM.

		Patients with contralateral CLNM
	Contralateral pretracheal lymph node-positive	Contralateral pretracheal lymph node-negative	*P*	
	(*n* = 25)	(*n* = 12)		
Mean age, yrs	38.5 ± 12.3	37.6 ± 7.3	0.034
≥55 years, *n* (%)	4 (16.0)	0	0.142^*^
Female, *n* (%)	17 (68.0)	8 (66.7)	0.935^*^
Primary tumor size (range), cm	1.49 ± 0.65	1.58 ± 1.1	0.108
≥1 cm, *n* (%)	18 (72.0)	8 (66.7)	0.740^*^
Multifocality, *n* (%)	8 (32.0)	1 (8.3)	0.220^*^
Extrathyroidal extension, *n* (%)	2 (8.0)	3 (25.0)	0.251^*^
Prelaryngeal LNM, *n* (%)	13 (52.0)	7 (58.3)	0.717
Ipsilateral CLNM, *n* (%)	22 (88.0)	8 (66.7)	0.183^*^
Ipsilateral pretracheal LNM, *n* (%)	18 (72.0)	5 (41.7)	0.146^*^

CLNM, central lymph node metastasis; LNM, lymph node metastasis. *Fisher’s exact test.

**Table 4:** Multivariate analysis of risk factors for contralateral CLNM.

	β (SE)	*P*	OR	95% CI (OR)	
				Lower	Upper
Prelaryngeal LNM	1.240 (0.430)	0.004	3.457	1.489	8.026
Contralateral pretracheal LNM	1.213 (0.445)	0.006	3.362	1.407	8.037
Ipsilateral CLNM	0.758 (0.500)	0.13	2.133	0.8	5.685
Constant	-2.665 (0.500)				

CI, confidence interval; CLNM, central lymph node metastasis; LNM, lymph node metastasis; OR, odds ratio; SE, standard error.

### Postoperative Morbidities and RAI Therapy

Forty-five (32.4%) of 139 patients developed postoperative hypocalcemia and they all recovered within 6 months. Five patients developed hoarseness and were confirmed to have vocal cord palsy by fibrolaryngoscopic examination. Four of the 5 patients recovered to normal cord mobility within 6 months. Forty-nine (35.3%) patients received RAI therapy (100–150 mCi) after surgery, and no patient had neck recurrence (central or lateral) or distant metastasis during the mean follow-up duration of 9.1 ± 1.8 months (range, 6–20 months).

## Discussion

In most patients with PTC, cervical nodal metastasis generally occurs in a stepwise sequential fashion (12). Tumors located in the upper part of the thyroid and the pyramidal lobe usually first metastasize to prelaryngeal and carotid triangular LNs, and those located in the middle and lower parts of the thyroid generally first metastasize to ipsilateral CLNs or pretracheal LNs. To the best of our knowledge, the majority of published studies reported that the region ipsilateral to the tumor is the most common metastatic region (2, 13, 14). In contrast, the results of this study showed that the subsite with the highest rate of LNM in the central neck was the pretracheal region (73.4%), followed by the ipsilateral paratracheal region (63.3%).

A few studies have described the pros and cons of prophylactic CND. Some endocrine surgeons recommended performing bilateral CND as comprehensive dissection of CLNs may reduce the risk of locoregional recurrence and facilitate achieving low Tg levels (1, 15–17). In contrast, others performed unilateral CND because most patients with unilateral PTC have a lower rate of contralateral CLNM. They argued that bilateral CND did not significantly improve survival but may increase the risk of postoperative morbidities (18, 19). The incidence of LNM to the contralateral paratracheal region in cN0 PTC patients has been reported to range from 9.8% to 30.6% (20–23). In the present study, we found that the rate of contralateral CLNM was 26.6% (37/139). Our findings also demonstrated that the incidence of contralateral CLNM was relatively low in unilateral cN0 PTC patients. Thus, the surgical extent of ipsilateral CND is sufficient for most unilateral PTCs.

However, ipsilateral CND means that there is a potential risk of locoregional recurrence in the contralateral paratracheal region. An accurate assessment of cervical lymph node status is the key to reducing the risk of locoregional recurrence and surgical complications. Preoperative US is helpful in detecting metastatic LNs in the neck, but the sensitivity is relatively low because of the overlying thyroid gland and trachea (24). In fact, a large proportion of PTC patients with node negative on preoperative US or palpation were found to have cervical LNM on pathological examination after surgery (24, 25). A few studies have suggested some risk factors for predicting contralateral CLNM in cN0 PTC (13, 14, 20–22). A multicenter study reported that contralateral CLNM was associated with ipsilateral paratracheal LNM (20). In another study, pretracheal and prelaryngeal LNM independently predicted contralateral CLNM (26). The results of this study also showed that patients with prelaryngeal LN involvement had a significantly higher rate of contralateral CLNM, and prelaryngeal LNM was an independent risk factor for contralateral CLNM. Recently, some endocrine surgeons have further investigated the value of intraoperative re-evaluation of prelaryngeal and pretracheal LNs through frozen section examination (FSE) in the decision-making of bilateral CND and found that FSE is a safe and effective strategy to decrease the need for a second-step CND (27, 28).

Theoretically, the risk of contralateral CLNM will increase when pretracheal LNs are positive. However, in our study, pretracheal LN metastasis was not associated with contralateral CLNM. In contrast, contralateral pretracheal LNM was significantly associated with contralateral CLNM, and contralateral pretracheal LNM was an independent predictor for contralateral CLNM. Our data suggest that pretracheal LN subdivision has potential value for clinical application, which is helpful for surgeons to determine the extent of CND. To the best of our knowledge, this was the first study assessing contralateral pretracheal LNM in predicting contralateral CLNM in unilateral cN0 PTC. Based on the results of our study, contralateral pretracheal LNs could be considered sentinel compartments in bilateral CND, and intraoperative FSE should be considered and performed. Locoregional recurrence after CND remains a critical issue in PTC, although the prognostic relevance of microscopic CLNM to recurrence is likely minimal in most cases. Unfortunately, we were unable to determine whether prophylactic contralateral CND is essential in patients with microscopic CLNM since there was a lack of comparison of the cohort group and a short follow-up period in this study. Therefore, we suggest that PTC patients with unilateral tumors presenting with evident nodal disease intraoperatively or preoperatively in the contralateral central neck should undergo therapeutic bilateral CND. Otherwise, ipsilateral CND may be appropriate.

It is evident that there are several limitations in this study. First, although it is conducted prospectively, a small sample size without comparative analysis cannot be overlooked. Further prospective, randomized trials with large sample size are warranted to validate the value of this intraoperative decision-making approach. Second, a longer follow-up duration is required to assess locoregional recurrence and survival. In spite of these limitations, our study has the merit of including cN0 PTC patients operated on with the same therapeutic protocol in the same institution.

## Conclusions

Patients with unilateral PTC are at a relatively low risk of contralateral CLNM and CND limited to the ipsilateral side is appropriate. The results of the present study suggest that prelaryngeal LNM or contralateral pretracheal LNM increases the likelihood of contralateral CLNM. Therefore, intraoperative assessment of prelaryngeal and contralateral pretracheal LNs by using FSE is able to decide whether to perform contralateral CND in patients with unilateral PTC.

## Data Availability Statement

The raw data supporting the conclusions of this article will be made available by the authors, without undue reservation.

## Ethics Statement

The studies involving human participants were reviewed and approved by the Institutional Review Board of West China Hospital, Sichuan University. The patients/participants provided their written informed consent to participate in this study.

## Author Contributions

JZ, designed and conceptualized the study. AS, FL and JZ, conducted the study. WL and LZ, collected the data. QC and YL, data analysis and manuscript writing. JZ and AS, interpreted data and refined the manuscript. All authors contributed to the study and approved the submitted version.

## Conflict of Interest

The authors declare that the research was conducted in the absence of any commercial or financial relationships that could be construed as a potential conflict of interest.

## Publisher’s Note

All claims expressed in this article are solely those of the authors and do not necessarily represent those of their affiliated organizations, or those of the publisher, the editors and the reviewers. Any product that may be evaluated in this article, or claim that may be made by its manufacturer, is not guaranteed or endorsed by the publisher.
